# TGF‐β isoforms inhibit hepatitis C virus propagation in transforming growth factor beta/SMAD protein signalling pathway dependent and independent manners

**DOI:** 10.1111/jcmm.16432

**Published:** 2021-03-08

**Authors:** Li‐Li Zou, Jian‐Rui Li, Hu Li, Jia‐Li Tan, Mei‐Xi Wang, Nan‐Nan Liu, Rong‐Mei Gao, Hai‐Yan Yan, Xue‐Kai Wang, Biao Dong, Yu‐Huan Li, Zong‐Gen Peng

**Affiliations:** ^1^ CAMS Key Laboratory of Antiviral Drug Research Institute of Medicinal Biotechnology Chinese Academy of Medical Sciences and Peking Union Medical College Beijing China; ^2^ Key Laboratory of Biotechnology of Antibiotics The National Health and Family Planning Commission (NHFPC) Institute of Medicinal Biotechnology Chinese Academy of Medical Sciences and Peking Union Medical College Beijing China; ^3^ Beijing Key Laboratory of Antimicrobial Agents Institute of Medicinal Biotechnology Chinese Academy of Medical Sciences and Peking Union Medical College Beijing China

**Keywords:** addition, hepatitis C virus, liver fibrosis, TGF‐β isoform, TGF‐β signalling pathway

## Abstract

Transforming growth factor beta (TGF‐β) plays an important role in the viral liver disease progression via controlling viral propagation and mediating inflammation‐associated responses. However, the antiviral activities and mechanisms of TGF‐β isoforms, including TGF‐β1, TGF‐β2 and TGF‐β3, remain unclear. Here, we demonstrated that all of the three TGF‐β isoforms were increased in Huh7.5 cells infected by hepatitis C virus (HCV), but in turn, the elevated TGF‐β isoforms could inhibit HCV propagation with different potency in infectious HCV cell culture system. TGF‐β isoforms suppressed HCV propagation through interrupting several different stages in the whole HCV life cycle, including virus entry and intracellular replication, in TGF‐β/SMAD signalling pathway–dependent and TGF‐β/SMAD signalling pathway–independent manners. TGF‐β isoforms showed additional anti‐HCV activities when combined with each other. However, the elevated TGF‐β1 and TGF‐β2, not TGF‐β3, could also induce liver fibrosis with a high expression of type I collagen alpha‐1 and α‐smooth muscle actin in LX‐2 cells. Our results showed a new insight into TGF‐β isoforms in the HCV‐related liver disease progression.

## 1 INTRODUCTION

Transforming growth factor beta (TGF‐β) isoforms, including TGF‐β1, TGF‐β2 and TGF‐β3, are 25‐kD secreted homodimeric signalling proteins.^1^ TGF‐β isoforms exert their biological effects through initiating the TGF‐β signalling. Two of mature TGF‐β constitute a homodimer and then bind to transmembrane receptors TGF‐β receptor type I (TβRI) and TGF‐β receptor type II (TβRII) to form a heterotetramer, where TβRI is phosphorylated by TβRII. The phosphorylated TβRI not only activates Smad protein (SMAD)‐independent cascades but also promotes SMAD‐dependent cascades by phosphorylating receptor‐regulated SMADs (including SMAD2 and SMAD3), which subsequently form active SMAD complexes with SMAD4 and regulate the transcription of target genes, such as type I collagen alpha‐1 (COL1A1), vascular endothelial growth factor A (VEGFA) and interleukin‐17A (IL‐17A).^1^, ^2^, ^3^, ^4^ Recently, accumulated evidence indicated that activated TGF‐β signalling is involved in various cellular processes, such as cell recognition, differentiation, proliferation and apoptosis.^3^ However, the role of different TGF‐β isoforms in the development of liver disease induced by hepatitis C virus (HCV) infection and the underlying mechanisms remain largely unclear.

**FIGURE 1:**
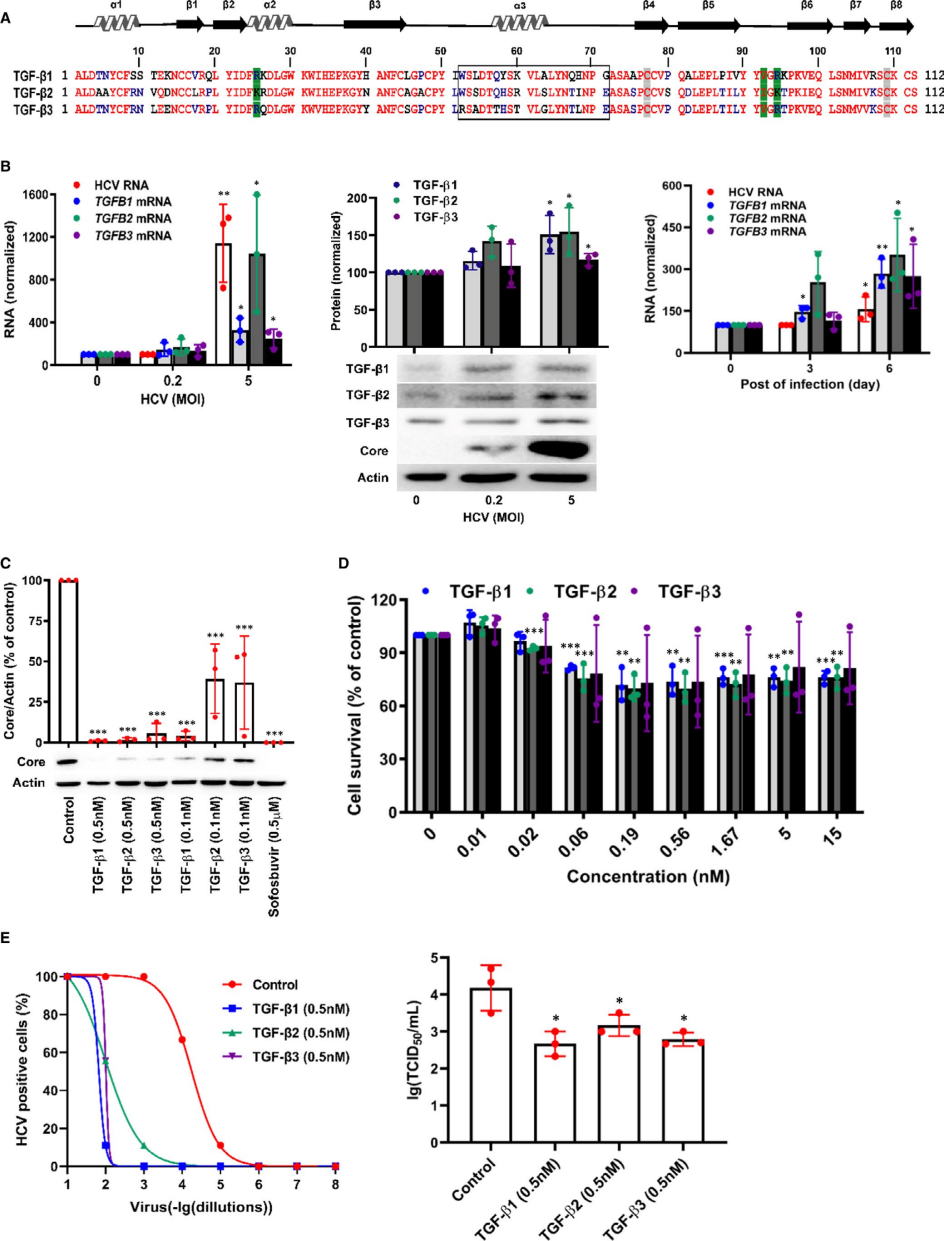
TGF‐β isoforms inhibit HCV propagation in HCVcc system. A, Sequence alignment of C‐terminal domains of mature human TGF‐β isoforms. Green shading represented the amino acid residues contacting with TβRII; grey shading represented the cysteine residues that formed the inter‐chain disulphide; open box designated the residues of α‐helix 3 in TGF‐β isoforms; the homology sequence of TGF‐β isoforms was indicated in red text. B, Huh7.5 cells were treated with HCV at different MOI for 72 h or at MOI = 0.2 for 0, 3 days or 6 days, and then TGF‐β isoforms mRNA or protein were quantified by qRT‐PCR or WB analysis. Huh7.5 cells were infected with HCV for 72 h in the presence of TGF‐β isoforms or sofosbuvir. Intracellular proteins were extracted and detected by WB (C) and the cytotoxicity without HCV infection was identified using an MTT assay (D). E, Huh7.5 cells were incubated with HCV viral stock at 10‐fold dilutions ranged from 10^−1^ to 10^−8^, and simultaneously treated with 0.5 nmol/L of TGF‐β1, TGF‐β2 and TGF‐β3. At 72 h, HCV‐positive wells were examined using a microscope after immunostaining against HCV core protein (left). Huh7.5 cells were inoculated with HCV viral stock and 0.5 nmol/L of TGF‐β1, TGF‐β2 or TGF‐β3 for 24 h. The newly released infectious virus particles were collected at 48 h and then quantified by TCID_50_ assay (right). Data represent the mean ± SD of three independent experiments. ANOVA analysis followed by the *Student's t* test method was used. *P < 0.05, **P < 0.01, ***P < 0.001 vs the control group

In the liver tissue, TGF‐β isoforms are produced and secreted not only by non‐parenchymal cells but also by hepatocytes. The expressions of TGF‐β isoforms are promoted by HCV infection in cultured hepatocytes and in livers of HCV‐infected patients, which might be caused by HCV‐induced endoplasmic reticulum stress, unfolded protein response activation or HCV core expression.^5^, ^6^, ^7^, ^8^ Although TGF‐β plays essential roles in the liver disease progression, including initial liver injury, fibrosis, cirrhosis and hepatocellular carcinoma,^9^ it is demonstrated that TGF‐β suppressed HCV RNA replication and protein expression depending on TGF‐β/SMAD signalling pathway in HCV sub‐genomic replicon system, and TGF‐β1 could directly suppress hepatitis B virus (HBV) replication in the cell culture system.^10^, ^11^ Furthermore, though the three isoforms of TGF‐β exhibit significant sequence homology (71%–79% identity, Figure 1A), they have opposite effects on fibrosis disease progression, with TGF‐β1 promoting fibrosis while TGF‐β3 having anti‐fibrotic effect.^1^, ^2^, ^6^, ^12^ Here, we investigated the antiviral activities and the underlying mechanisms of TGF‐β isoforms by which they inhibited HCV propagation.

## 2 MATERIALS AND METHODS

### 2.1 Cell line and virus

The human hepatoma cell line Huh7.5 cells and the plasmid pFL‐J6/JFH/JC1 (HCV‐2a) containing the full‐length chimeric HCV complementary DNA (cDNA) were kindly provided by Vertex Pharmaceuticals Inc.^13^ The cells were cultured in Dulbecco's modified Eagle medium (DMEM, Gibco) supplemented with 10% heat‐inactivated foetal bovine serum (FBS, Gibco) and 1% penicillin‐streptomycin (PS, Gibco). Infectious HCV virus stock was prepared as previously described.^13^ Huh7.5 cells, GS4.3 replicon cells and LX‐2 cells were cultured as described before.^14^, ^15^

### 2.2 Reagents and antibodies

TGF‐β1 (100‐21‐10 μg), TGF‐β2 (100‐35B‐10 μg) and TGF‐β3 (100‐36E‐10 μg) proteins were purchased from PeProtech Inc (Peprotech). All TGF‐β isoforms were dissolved according to the manufacturer's protocol and the cell viability after TGF‐β isoforms treatment was determined by a methyl thiazolyl tetrazolium (MTT, Amresco) assay.^14^ Sofosbuvir (HY‐15005), TβRI/II inhibitor LY2109761 (HY‐12075) and TβRI/ALK5 inhibitor RepSox (HY‐13012) were purchased from MedChem Express (MCE). The following primary antibodies were used: anti‐HCV core (ab2740, Abcam), anti‐NS3 (ab13830, Abcam), anti‐β‐Actin (3700S, Cell Signaling Technology), anti‐TGF‐β1 (ab179695, Abcam), anti‐TGF‐β3 (ab15537, Abcam), anti‐TβRI (ab121024, Abcam), anti‐SMAD2/3 (8685T, Cell Signaling Technology) and corresponding secondary antibodies (ZSGB‐BIO). The siRNAs for TβRI (sc‐40222) and for SMAD2/3 (sc‐37238) and the negative control siRNA‐A (sc‐37007) were purchased from Santa Cruz Biotechnology.

### 2.3 Plasmids

The plasmids pcDNA3.1(+)‐TGF‐β1 expressing TGF‐β1, pcDNA3.1(+)‐TGF‐β3 expressing TGF‐β3, pcDNA3.1(+)‐TGF‐β1‐His expressing TGF‐β1 with His tag at the C‐terminal, pcDNA3.1(+)‐TGF‐β3‐His expressing TGF‐β3 with His tag at the C‐terminal and the part truncated mutant plasmids of pcDNA3.1(+)‐TGF‐β1‐His or pcDNA3.1(+)‐TGF‐β3‐His were generated by amplification of cDNA from Huh7.5 cell using primers shown in Table 1. The sequences were assessed by assay of restriction endonucleases and sequencing. All mutagenesis was performed by Taihe Biotechnology (China).

**TABLE 1:** The oligonucleotide sequences used in PCR or qRT‐PCR

Gene	Sequence (5'→3')	
*For plasmid*		
TGF‐β1	S	CCGGAATTCCGGATGCCGCCCTCCGGGCTGCG
	R	GCTCTAGAGCTCAGCTGCACTTGCAGGAGC
TGF‐β3	S	CCCAAGCTTATGAAGATGCACTTGCAAAGGGCTC
	R	CCGCTCGAGTCAGCTACATTTACAAGACTTCACCACCA
TGF‐β1‐His	S	GGAATTCCATGCCGCCCTCCGGGCTGCG
	R	CTCTAGATCAATGGTGATGGTGATGATGGCTGCACTTGCAGGAGCGCA
TGF‐β3‐His	S	CCCAAGCTTATGAAGATGCACTTGCAAAGGGCTC
	R	CCGCTCGAGTCAATGGTGATGGTGATGATGGCTACATTTACAAGACTTCACCACCA
TGF‐β1△α3‐His	S1	GCACAGTGGCGGCCGCATGCCGCCCTCCGGGCTGCG
	R1	GCCGAGGCAATGTAGGGGCAGGGCCCGAGGCAGAAG
	S2	CCTACATTGCCTCGGCGGCGCCGTGCTGCGTGCCGC
	R2	GGCCCTCTAGACTCGATCAATGGTGATGGTGATGATGGCTGCACTTGCAGGAGCG
TGF‐β3△α3‐His	S1	GCACAGTGGCGGCCGCATGAAGATGCACTTGCAAAG
	R1	GCAGATGCGAGGTATGGGCAAGGGCCTGAGCAGAAG
	S2	CATACCTCGCATCTGCCTCGCCTTGCTGCGTGCCCC
	R2	GGCCCTCTAGACTCGATCAATGGTGATGGTGATGATGGCTACATTTACAAGACTTC
*For qRT‐PCR*		
HCV	S	CGGGAGAGCCATAGTGGTCTGCG
	R	CTCGCAAGCACCCTATCAGGCAGTA
	P	FAM‐5′‐AGGCCTTGTGGTACTGCCT‐3′‐TAMRA
GAPDH	S	CGGAGTCAACGGATTTGGTCGTAT
	R	AGCCTTCTCCATGGTGGTGAAGAC
	P	FAM‐5′‐CCGTCAAGGCTGAGAACGG‐3′‐TAMRA
TGF‐β1	S	GCAGCACGTGGAGCTGTA
	R	CAGCCGGTTGCTGAGGTA
TGF‐β2	S	CCTTCTTCCCCTCCGAAAC
	R	AGAGCACCTGGGACTGTCTG
TGF‐β3	S	CGCAGTGCAGACACAA
	R	TGCTTCAGGGTTCAGA
COL1A1	S	TCTGGCGCTCCCATGGCTCT
	R	GCCCTGCGGCACAAGGGATT
α‐SMA	S	GAGTTACGAGTTGCCTGATGG
	R	GATGCTGTTGTAGGTGGTTTCA

Abbreviations: P, probe; R, reverse complementary sequence; S, sense sequence.

### 2.4 Quantification of HCV RNA and mRNA

The cellular RNAs harvested by RNeasy Mini Kit (QIAGEN) from Huh7.5 cells were detected using the AgPath‐ID One‐Step RT‐PCR Kit (Applied Biosystems). Fluorescent signals were identified with 7500 fast real‐time PCR system (Applied Biosystems) according to the procedure recommended by the manufacturer. HCV RNA level was calculated according with the 2^−ΔΔCT^ method. Glyceraldehyde 3‐phosphate dehydrogenase (*GAPDH*) was served as the internal control for the quantification. The primer and probe sequences used for quantitative real‐time reverse transcriptase polymerase chain reaction (qRT‐PCR) are provided in Table 1.

For mRNA quantitation by SYBR Green method, total RNAs were reverse‐transcribed with PrimeScript RT Master Mix (TaKaRa, Japan), and the mRNAs of *TGFB1*, *TGFB2*, *TGFB3*, type I collagen alpha 1 (*COL1A1*) or alpha‐smooth muscle actin (*α‐SMA*) were quantified using GoTaq qPCR Master Mix according to the procedure. The primers are shown in Table 1.

### 2.5 Induction of gene expression of TGF‐β isoforms by HCV infection

Huh7.5 cells were treated with HCV viral stock at different multiplicity of infection (MOI = 0, 0.2 and 5) for 72 hours or at MOI = 0.2 for 0, 3 days or 6 days, and then, intracellular mRNAs or proteins were extracted and quantified by qRT‐PCR or Western blot (WB) analysis.

### 2.6 Determination of anti‐HCV activity

Huh7.5 cells were inoculated with HCV viral stock (MOI = 0.5) and simultaneously treated with TGF‐β isoforms or sofosbuvir. At 72 hours, intracellular proteins were harvested by the CytoBuster Protein Extraction Reagent (PER, Novagen) containing 1 mmol/L protease inhibitor cocktail (Roche Applied Science) and HCV core protein level in cells was determined by WB analysis.

For measuring HCV infectivity, Huh7.5 cells were incubated with HCV viral stock at 10‐fold dilutions ranged from 10^−1^ to 10^−8^ and simultaneously treated with 0.5 nmol/L of TGF‐β1, TGF‐β2 and TGF‐β3. At 72 hours post‐infection, HCV‐positive wells were examined using a microscope after immunostaining against HCV core protein.^16^

To identify the effects of TGF‐β isoforms on *de novo* HCV particle production, Huh7.5 cells were inoculated with HCV viral stock (MOI = 0.7) and 0.5 nmol/L of TGF‐β1, TGF‐β2 or TGF‐β3 for 24 hours. The newly released infectious virus particles were collected at 48 hours and then quantified by 50% tissue culture infective dose (TCID_50_) assay. The infectivity titre was shown as TCID_50_ per mL.^17^, ^18^, ^19^

GS4.3 cells were treated with 0.5 nmol/L of TGF‐β isoforms or sofosbuvir (0.5 μmol/L) for 72 hours. Then, intracellular proteins were extracted by PER and HCV NS3 protein level was detected by WB analysis.

### 2.7 HCV entry inhibition and time‐of‐addition assays

To evaluate the antiviral effective stages of TGF‐β isoforms, Huh7.5 cells were pre‐treated with TGF‐β isoforms (0.5 nmol/L) for 2 hours followed by HCV infection for 2 hours, simultaneously treated with TGF‐β isoforms (0.5 nmol/L) and HCV for 2 hours, or infected with HCV (MOI = 0.5) for 2 hours followed by TGF‐β isoforms (0.5 nmol/L) 2 hours treatment. At 72 hours, intracellular HCV core protein level was detected by WB analysis. In order to further characterize the inhibition stages, a time‐of‐addition assay was performed. In brief, Huh7.5 cells were inoculated with HCV (MOI = 0.5) at 37°C for 2, 4, 6, 8 hours or 10 hours, and then, the HCV‐containing medium was replaced by TGF‐β isoforms (0.5 nmol/L) or sofosbuvir (0.5 μmol/L) containing medium. After 2 hours of treatment, the medium was replaced by fresh medium. At 72 hours, HCV core protein level in cells was determined by WB analysis.

### 2.8 Inactivation viral particle assay

HCV viral stock (MOI = 0.5) was pre‐treated with 0.2 nmol/L TGF‐β1, 0.5 nmol/L TGF‐β2 or 0.5 nmol/L TGF‐β3 at 37°C and used to infect cells after 25‐fold dilution of the inoculum. Infection with untreated HCV stock was performed in parallel in the presence of TGF‐β isoforms at indicated concentrations. At 72 hours, intracellular proteins were harvested and HCV core protein level was determined by WB.

### 2.9 The inhibitory activities of TGF‐β isoforms on HCV particle release

Huh7.5 cells were incubated with HCV viral stock (MOI = 0.5) for 16 hours, and then, the cells were treated with TGF‐β isoforms (0.5 nmol/L), sofosbuvir (0.5 μmol/L) or 0.1% BSA for 48 hours. The culture medium was replaced by adding fresh culture medium. After 24 hours, the cell viability was tested using MTT assay, the culture medium was collected for incubation to naïve Huh7.5 cells, and intracellular RNA was extracted and quantified by qRT‐PCR. Meanwhile, naïve Huh7.5 cells were incubated with above culture supernatants for 72 hours. Then, the intracellular RNA of Huh7.5 cells was extracted and quantified by qRT‐PCR.

### 2.10 The combined antiviral activities of TGF‐β isoforms

Huh7.5 cells were inoculated with HCV viral stock (MOI = 0.5) and simultaneously treated with 0.04 nmol/L TGF‐β1, 0.05 nmol/L TGF‐β2, 0.1 nmol/L TGF‐β3 alone, in combination or double concentration (2×), or sofosbuvir (0.5 μmol/L). At 72 hours, intracellular proteins were extracted and determined by WB analysis. To evaluate their combined effect, the combination index (CI) showed with *q* value was calculated with the improved Bürgi formula (Jin's equation), with *q* < 0.85, 0.85 ~ 1.15, and >1.15 indicating antagonism, addition and synergy, respectively.^20^

### 2.11 The effect of TGF‐β receptor inhibitors on antiviral activities

Huh7.5 cells were incubated with TβRI or TβRII kinase inhibitors (20 μmol/L LY2109761 or 50 μmol/L RepSox) for 6 hours, and then, TGF‐β isoforms (0.5 nmol/L) and HCV (MOI = 0.5) were simultaneously added. After 72 hours incubation, intracellular HCV core protein was determined by WB analysis.

### 2.12 Overexpression and RNA interference

Huh7.5 cells were transfected with TGF‐β1, TGF‐β1‐His, TGF‐β3, TGF‐β3‐His, the mutants of TGF‐β plasmids or plasmid control pcDNA3.1(+) applying HD transfection reagent (Promega), or transfected with siRNA for TβRI or SMAD2/3 or negative control siRNA‐A applying RNAiMAX transfection reagent (Invitrogen). After 48 hours, the cells were sub‐cultured and part of intracellular proteins was harvested to detect His tag or corresponding protein expression with WB. After 24 hours, cells were infected with HCV (MOI = 0.5) or added TGF‐β isoforms (0.5 nmol/L) and HCV (MOI = 0.5). After 48 hours, intracellular proteins were extracted to detect HCV core protein level by WB.

### 2.13 Hepatic stellate cell activation of TGF‐β isoforms

LX‐2 cells were cultured in high glucose DMEM without FBS for 12 hours. Then, the cell supernatant was replaced by fresh medium with 2% FBS containing 0.5 nmol/L TGF‐β isoforms. After 24 hours, intracellular RNA was extracted and *COL1A1* and *α‐SMA* mRNA was quantified with qRT‐PCR.

### 2.14 Western blot analysis

The Western blot analysis was performed as previously described.^21^ In brief, equal amounts of protein from cellular lysates were subjected to SDS‐PAGE and electroblotted onto transmembrane (Millipore). Proteins were probed with antibodies against HCV core, TGF‐β1, TGF‐β3, His tag, TβRI or SMAD2/3, respectively. β‐Actin antibody was used as an internal control. Proteins were detected by chemiluminescence with Immobilon Western Chemiluminescent HRP Substrate (Millipore) and visualized by ChemiDo XRS gel imager system (Bio‐Rad). Protein band intensity was analysed by Gelpro32 software. The ratio of interested protein to internal control protein β‐Actin was calculated and normalized as 1.00 for the control group.

### 2.15 Statistical analysis

The data were presented as means ± standard deviation (SD) more than three independent experiments. Statistical analyses were performed in GraphPad Prism 7 (GraphPad) using ANOVA analysis followed by the Student’s *t* test; *P* < 0.05 was considered as statistically significant.

## 3 RESULTS

### 3.1 TGF‐β isoforms inhibit HCV propagation in vitro

In infectious HCV cell culture (HCVcc) system, the three TGF‐β isoforms at mRNA (Figure 1B, left) and protein (Figure 1B, middle) levels were all elevated after 3 days of HCV infection in an infectious dose‐dependent manner, and the TGF‐β isoforms at mRNA levels were also increased in a time‐dependent manner at the low MOI of 0.2 (Figure 1B, right). The results agree with previous reports showing that in situ expressions of TGF‐β isoforms in the liver of HCV‐infected patients and the increased mRNA and protein expressions of TGF‐β1 and TGF‐β2 in HCV‐infected hepatocytes.^6^, ^7^, ^8^ Moreover, after HCV infection the increased expressions of TGF‐β2 at mRNA and protein levels were relatively higher compared to TGF‐β1 and TGF‐β3 (Figure 1B), which was consistent with previous studies.^4^, ^5^, ^6^, ^7^, ^8^, ^22^

Then, we confirmed whether TGF‐β isoforms still have the anti‐HCV activities in HCVcc system. Following HCV infection and treatment of different concentrations of TGF‐β isoforms for 72 hours, the level of HCV core protein in Huh7.5 cells was quantified with WB analysis. The results showed that all of the three TGF‐β isoforms significantly reduced HCV core protein level in a dose‐dependent manner with TGF‐β1 possessing the best anti‐HCV effect (Figure 1C), though a slightly detectable cytotoxicity was detected with an MTT assay without a significant dose‐dependent manner (Figure 1D), which might be induced by the inhibition of TGF‐β to tumour cells.^23^ To further confirm the anti‐HCV activities of TGF‐β isoforms, we analysed the HCV infectivity after TGF‐β isoforms treatment using immunostaining assay. After 3 days of treatment with TGF‐β isoforms, HCV‐positive cells were reduced (Figure 1E, left), hinting that TGF‐β isoforms could disable HCV propagation. Then, to identify the effects of TGF‐β isoforms on *De novo* HCV production, Huh7.5 cells were infected with HCV and simultaneously treated with TGF‐β isoforms for 24 hours, and newly released infectious virus particles were collected at 48 hours and then quantified by TCID_50_ assay. *De novo* produced HCV yielded a viral titre of about 10^4.17^ TCID_50_ per mL, whereas treatment with TGF‐β isoforms significantly decreased the TCID_50_ of the *De novo* produced HCV (Figure 1E, right). The results suggested that HCV infection induced TGF‐β isoforms expression, and in turn, the elevated TGF‐β isoforms could down‐regulate HCV propagation.

### 3.2 The distinct anti‐HCV mechanisms of TGF‐β isoforms lead to their additional anti‐HCV effects

**FIGURE 2:**
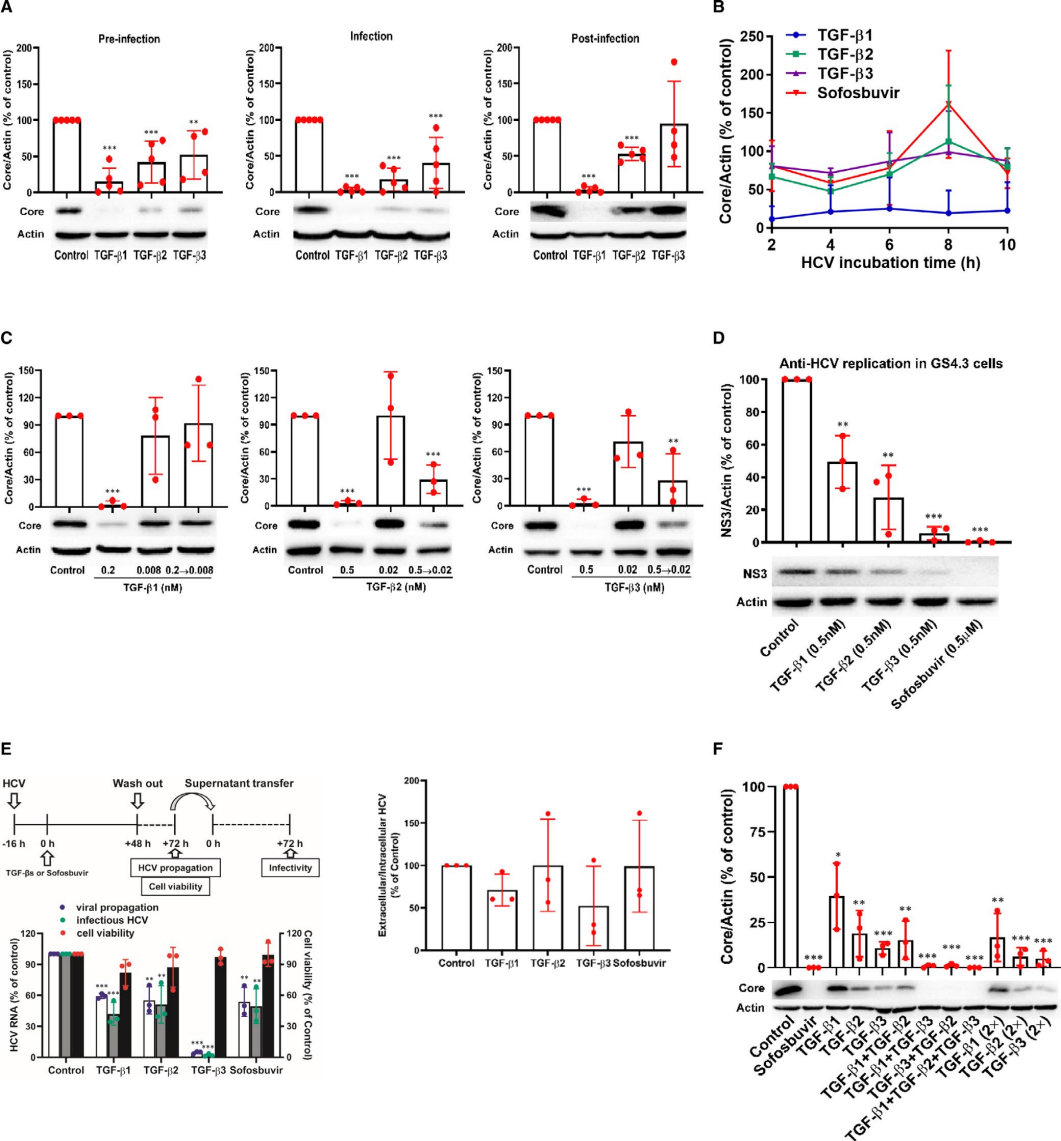
TGF‐β isoforms inhibit HCV at distinct stages in the HCV life cycle and have additional anti‐HCV effects combined with each other. A, Huh7.5 cells were pre‐treated with TGF‐β isoforms (0.5 nmol/L) for 2 h followed by HCV infection for 2 h (pre‐infection), simultaneously treated with TGF‐β isoforms (0.5 nmol/L) and HCV for 2 h (infection), or infected with HCV for 2 h followed by TGF‐β isoforms (0.5 nmol/L) 2 h treatment (post‐infection). Intracellular proteins were measured by WB analysis at 72 h. B, Huh7.5 cells were treated with TGF‐β isoforms (0.5 nmol/L) or sofosbuvir (0.5 μmol/L) for 2 h after 2, 4, 6, 8 and 10 h of HCV infection, intracellular proteins were quantified by WB at 72 h. C, HCV viral stock was pre‐treated with 0.2 nmol/L TGF‐β1, 0.5 nmol/L TGF‐β2 or 0.5 nmol/L TGF‐β3 at 37°C for 2 h and then used to infect Huh7.5 cells after 25‐fold dilution (the concentration of TGF‐β1 from 0.2 to 0.008 nmol/L, or TGF‐β2 or TGF‐β3 from 0.5 to 0.02 nmol/L). Infection with untreated HCV stock was performed in parallel in the presence of TGF‐β isoforms at indicated concentrations. Intracellular HCV core was determined by WB. D, GS4.3 cells were treated with TGF‐β isoforms or sofosbuvir for 72 h, and intracellular proteins were extracted and detected with WB. E, Schematic of the experiment (left, up). Huh7.5 cells were incubated with HCV viral stock for 16 h, and then treated with TGF‐β isoforms (0.5 nmol/L) or sofosbuvir (0.5 μmol/L) for 48 h followed by replacing the culture medium. After 24 h, the cell viability was tested using MTT assay, the culture medium was collected, and intracellular RNA was quantified by qRT‐PCR. Meanwhile, naïve Huh7.5 cells were incubated with above culture supernatants for 72 h and the intracellular RNA was quantified by qRT‐PCR (left, down). The ration of extracellular infective HCV particle and intracellular HCV level was calculated (right). F, Huh7.5 cells were inoculated with HCV viral stock and simultaneously treated with 0.04 nmol/L TGF‐β1, 0.05 nmol/L TGF‐β2, 0.1 nmol/L TGF‐β3 alone, in combination, double concentration (2×) or sofosbuvir (0.5 μmol/L). At 72 h, intracellular proteins were detected by WB analysis. *P < 0.05, **P < 0.01, ***P < 0.001 vs solvent control. Data represent the mean ± SD from more than three independent experiments. ANOVA analysis followed by the *Student's t* test method was used

To investigate which HCV viral cycle was interrupted by TGF‐β isoforms, the kinetics of HCV infection was assessed in the presence of TGF‐β isoforms. Huh7.5 cells were treated with the TGF‐β isoforms 2 hours before, during or post‐HCV infection for 2 hours. The maximum inhibitory effects of TGF‐β2 and TGF‐β3 were observed when they were added during HCV infection for 2 hours compared with pre‐infection and post‐infection (Figure 2A), suggesting their anti‐HCV effects mainly acted on virus entry stage. However, TGF‐β1 inhibited HCV propagation in the whole viral life cycle, including before, during and after virus entry (Figure 2A), while TGF‐β2, except TGF‐β3, inhibited HCV propagation after virus entry (Figure 2A).

To further explore the anti‐HCV effective stages of TGF‐β isoforms, we performed time‐of‐addition experiments by adding TGF‐β isoforms for 2 hours after 2, 4, 6, 8 and 10 hours of HCV infection. As shown in Figure 2B, TGF‐β1 showed similar HCV inhibition effects in all time‐of‐addition stages, which was consistent with our above results (Figure 2A). However, TGF‐β2 and TGF‐β3 treatment for 2 hours after 2, 4, 6, 8 and 10 hours of HCV infection had lower anti‐HCV activities than those of 72 hours treatment (Figure 1C). These results suggest that TGF‐β1 might down‐regulate HCV propagation at several stages of the whole HCV life cycle, while TGF‐β2 and TGF‐β3 might mainly interrupt HCV entry into cells.

We then detected whether TGF‐β isoforms inhibited HCV entry via direct action on viral particle. HCV virus stock was pre‐incubated with TGF‐β isoforms for 2 hours, respectively, and then inoculated to Huh7.5 cells after diluted pre‐incubation mixture 25 times. As a control, the untreated HCV performed in parallel in the presence of TGF‐β isoforms was also inoculated to Huh7.5 cells. The results exhibited TGF‐β2 and TGF‐β3, except TGF‐β1, had higher inhibitory effects following pre‐incubation with HCV prior to inoculation (Figure 2C), suggesting that TGF‐β2 and TGF‐β3 might directly act on HCV particles.

To validate whether TGF‐β isoforms might interrupt intracellular HCV replication after virus entry, we first applied GS4.3 cells, a human hepatoma Huh7 cell line, which carried an HCV sub‐genomic replicon I 377‐3’del.S.^24^ In replicon cells, the three TGF‐β isoforms (0.5 nmol/L) inhibited HCV replication at protein level (Figure 2D) and TGF‐β3 showed the best inhibitory effect, which was equal to that of sofosbuvir (0.5 μmol/L), an NS5B polymerase inhibitor. These data demonstrated the TGF‐β isoforms also interrupted HCV propagation in viral replication stage, especially TGF‐β3 (Figure 2D), which was similar to the results from other HCV sub‐genomic replicon system cured MH14 cells.^10^ However, due to the low anti‐HCV capacities of TGF‐β2 and TGF‐β3 after 2 hours treatment, we speculated TGF‐β2 and TGF‐β3 might indirectly interrupt HCV propagation in viral replication stage through indirect impacts, such as activating signalling pathways.

Then, we analysed the inhibitory effects of TGF‐β isoforms on the production of infectious virion. After 16 hours of HCV incubation, the Huh7.5 cells were treated with TGF‐β isoforms for 48 hours. The supernatants were replaced by fresh medium and then transferred to naïve Huh7.5 cells to measure viral infectivity at 72 hours (Figure 2E, left). Similarly, TGF‐β isoforms and sofosbuvir significantly decreased intracellular (Figure 2E, left, viral propagation) and extracellular (Figure 2E, left, infectious HCV) HCV levels with no or slightly cytotoxicity (Figure 2E, left, cell viability). However, when compared the ratio of extracellular and intracellular HCV levels with that of control, they all showed no significance (Figure 2E, right), suggesting the isoforms could not inhibit HCV particle secretion.

Furthermore, there was an interesting phenomenon. In Figure 2E, we investigated the inhibition effects of TGF‐β isoforms on intracellular viral propagation and extracellular infectious HCV by quantifying the HCV mRNA to identify the inhibitory activities of TGF‐β isoforms on HCV particle release. The results highlighted TGF‐β3 could more strongly inhibit HCV mRNA level than TGF‐β1 (Figure 2E). But in Figure 2A, we investigated which HCV viral cycle was interrupted by TGF‐β isoforms by analysing the anti‐HCV effects of TGF‐β isoforms with treatment for 2 hours. The data confirmed 2 hours pre‐treatment of TGF‐β1 showed the stronger anti‐HCV activities than TGF‐β3. The phenomenon revealed distinct anti‐HCV mechanisms of TGF‐β isoforms and the anti‐HCV activity of TGF‐β3 might depend on the treatment time.

All the above results suggested that TGF‐β isoforms could suppress HCV propagation at different stages of HCV life cycle. To further confirm that TGF‐β isoforms inhibiting HCV propagation can be through different mechanisms, we investigated their antiviral activities in combination. The combined treatment with TGF‐β isoforms could enhance the anti‐HCV effects (Figure 2F). When calculated with the improved Bürgi formula,^20^ the *q* value was 1.04 for combined treatment with TGF‐β1 and TGF‐β3, 1.01 with TGF‐β1 and TGF‐β2, and 0.92 with TGF‐β2 and TGF‐β3, respectively, suggesting that combined use of the three isoforms could additionally inhibit HCV propagation. Certainly, the anti‐HCV activity was also stronger in the group of combined treatment with the three isoforms (Figure 2F) and the additional concentration of each isoform also increased the anti‐HCV activities (Figure 2F). However, the anti‐HCV activity of the additional each isoform was relatively lower than that of the combined treatment (Figure 2F). Therefore, these data further highlighted that TGF‐β isoforms might exhibit their anti‐HCV activities through distinct mechanisms and could obtain an additional anti‐HCV effect.

### 3.3 TGF‐β/SMAD signalling pathway is essential for interrupting HCV propagation by TGF‐β1 and TGF‐β2, except TGF‐β3

**FIGURE 3:**
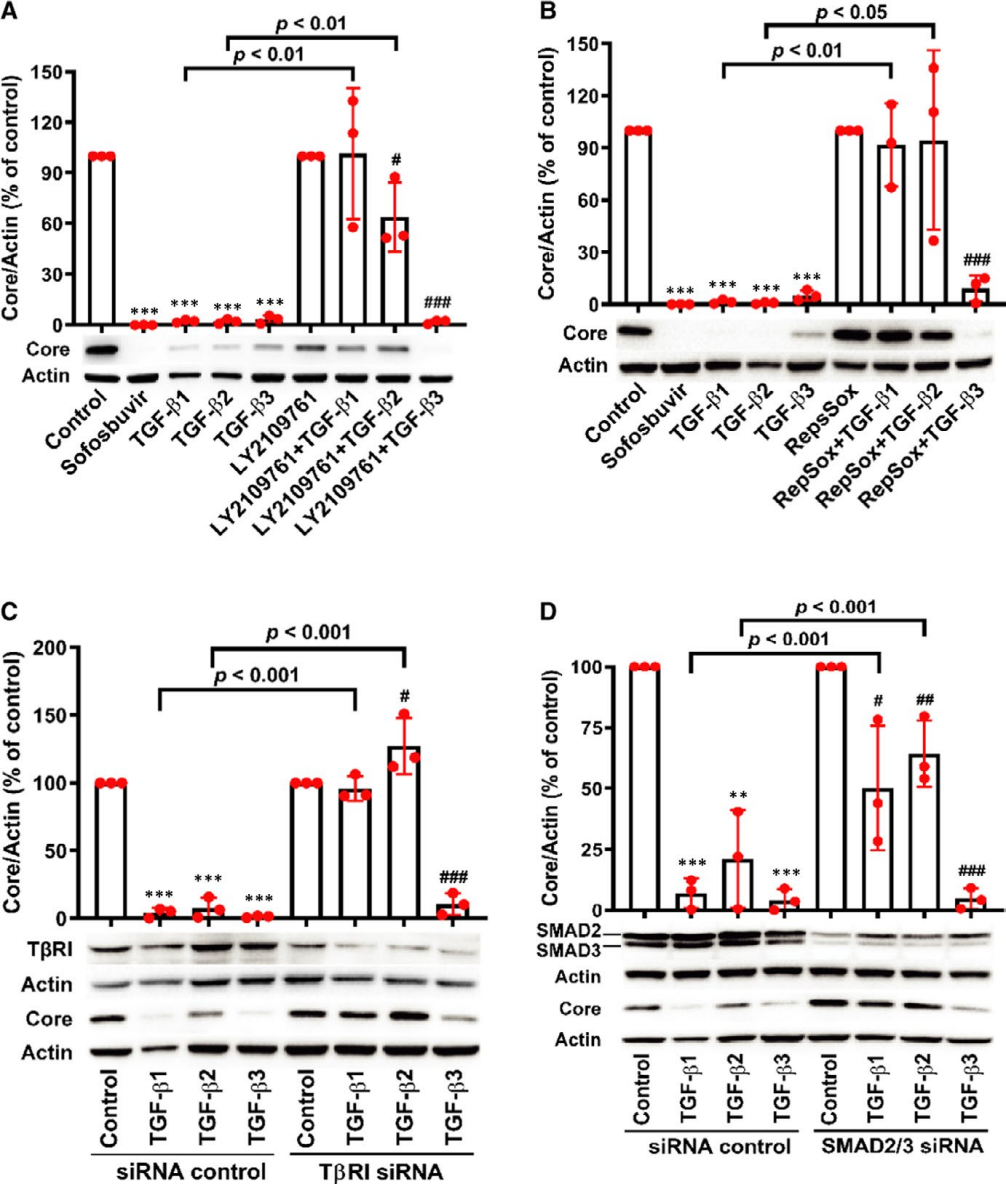
TGF‐β/SMAD signalling pathway is essential for anti‐HCV activities of TGF‐β1 and TGF‐β2, except TGF‐β3. HCV core level was determined by WB in Huh7.5 cells treated after 20 μmol/L LY2109761 (A) or 50 μmol/L Repsox (B) 6 h treatment prior to co‐incubation with 0.5 nmol/L of TGF‐β isoforms and HCV. At 72 h, intracellular HCV core was detected by WB. Huh7.5 cells were transfected with siRNA for TβRI (C) or siRNA for SMAD2/3 (D). After 48 h, the cells were sub‐cultured and then infected with HCV and simultaneously treated with 0.5 nmol/L TGF‐β isoforms after 24 h incubation. Intracellular HCV core was detected by WB after 48 h. **P < 0.01 and ***P < 0.001 vs solvent control or siRNA control plus solvent control group; ^#^ *P* < 0.05, ^##^ *P* < 0.01 and ^###^ *P* < 0.001 vs LY2109761 (Repsox or siRNA groups) control. Data represent the mean ± SD of three independent experiments. ANOVA analysis followed by the *Student's t* test method was used

Previous data suggested that the antiviral effect of TGF‐β might be associated with TGF‐β/SMAD signalling in HCV sub‐genomic replicon system.^10^ To further investigate whether TGF‐β/SMAD signalling was involved in anti‐HCV effects of TGF‐β isoforms in HCVcc system, we first used a serial of inhibitors. LY2109761, an orally active TβRI/II kinase dual inhibitor, dramatically reduced the anti‐HCV activities of TGF‐β1 and TGF‐β2, except TGF‐β3 (Figure 3A). Pre‐treatment with RepSox, an inhibitor of TβRI, also showed a significant reduction of the anti‐HCV effects of TGF‐β1 and TGF‐β2 with a negligible effect on TGF‐β3 (Figure 3B). Similarly, silencing TβRI in Huh7.5 cells with siRNA for TβRI also inhibited the anti‐HCV effects of TGF‐β1 and TGF‐β2, except TGF‐β3 (Figure 3C). Cumulatively, these data showed TβRII and TβRI, which is one of the key factors associated with TGF‐β/SMAD signalling pathway, played a pivotal role in anti‐HCV activities of TGF‐β1 and TGF‐β2 other than TGF‐β3.

To gain insight into the effect of TGF‐β/SMAD signalling pathway on the anti‐HCV activities of TGF‐β isoforms, we further used siRNAs against human SMAD2/3 to assess the contribution of SMAD2/3 to the anti‐HCV effects. Similarly, down‐regulation of SMAD2/3 protein expression significantly decreased the anti‐HCV capacities of TGF‐β1 and TGF‐β2, except TGF‐β3 (Figure 3D).

Those data highlighted that TGF‐β1 and TGF‐β2 might suppress HCV propagation through TGF‐β/SMAD signalling pathway, while TGF‐β3 inhibited HCV propagation independent on TGF‐β/SMAD signalling pathway.

### 3.4 Residues of Arg25, Val92 and Arg94 and dimer formation mediated by Cys77 or Cys109 residue are responsible for the anti‐HCV activities of TGF‐β isoforms independent of TGF‐β α‐helix 3

**FIGURE 4:**
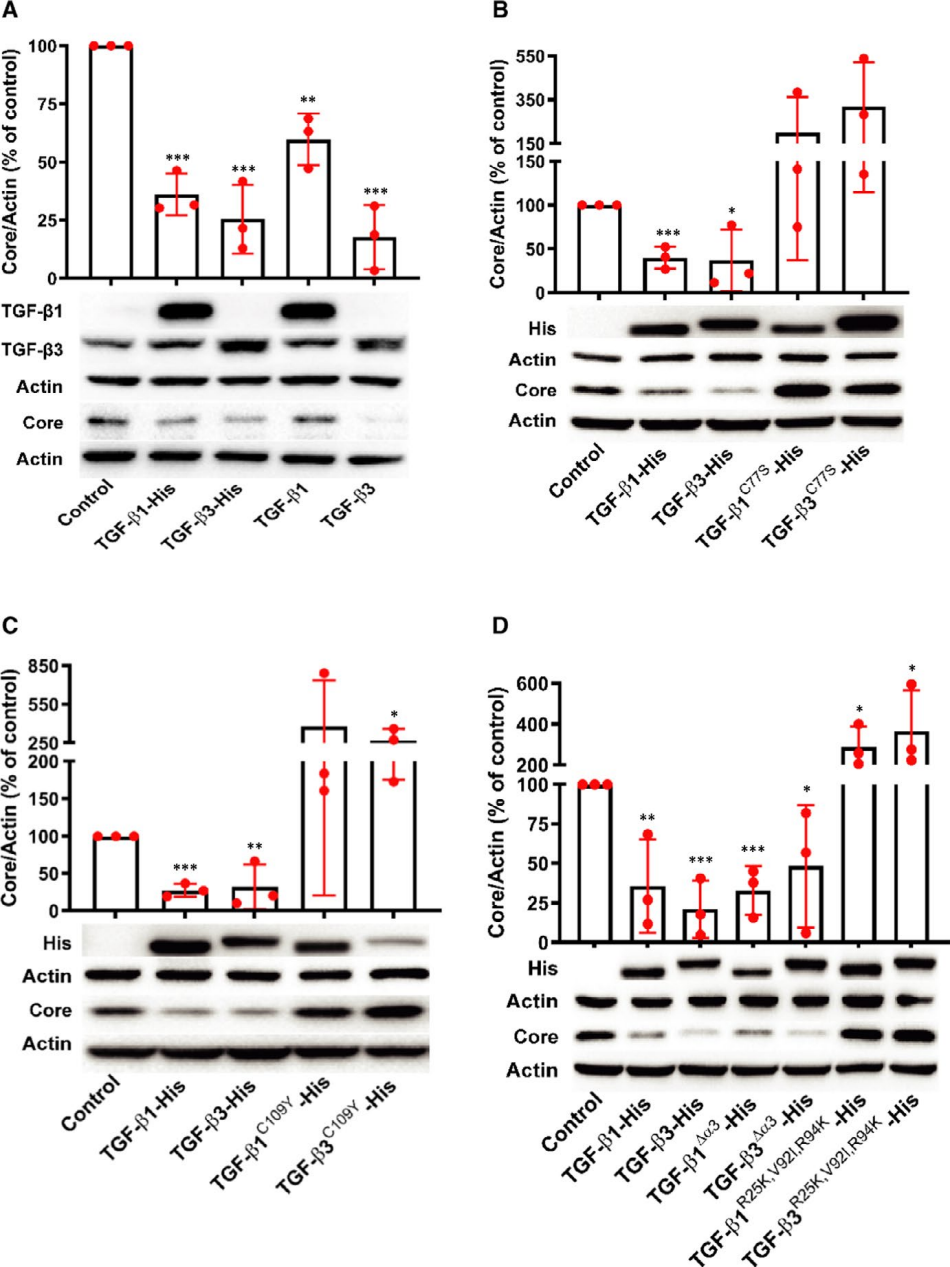
Anti‐HCV effects are eliminated by residue mutations in TGF‐β isoforms, but not influenced by deletion of α‐helix 3. Huh7.5 cells transfected with plasmids with His tag at C‐terminal (A), C77S mutant (B), C109Y mutant (C), TGF‐β α‐helix 3 deletion, or R25K, V92I and R94K mutants (D) for 48 h were sub‐cultured and then infected with HCV after 24 h. Intracellular HCV core was detected by WB after 48 h. Data represent the mean ± SD of three independent experiments. ANOVA analysis followed by the *Student's t* test method was used. *P < 0.05, **P < 0.01 and ***P < 0.001 vs plasmid control

In order to investigate which regions or amino acid residues of TGF‐β isoforms were responsible for their anti‐HCV capacities, we first compared the antiviral activities between overexpressed TGF‐β isoforms and with His tag at C‐terminal. However, we found the plasmid pcDNA3.1(+)‐TGF‐β2 could not well express TGF‐β2 in Huh7.5 cells as TGF‐β1 and TGF‐β3 did. Therefore, we only test the ant‐HCV effects caused by overexpressing TGF‐β1 and TGF‐β3 to investigate which regions or amino acid residues of TGF‐β isoforms were responsible for their anti‐HCV capacities. Overexpressing TGF‐β1 and TGF‐β3 with His tag at C‐terminal had equal anti‐HCV effects as TGF‐β1 and TGF‐β3, respectively (Figure 4A), suggesting that fusing His tag did not affect the functions of TGF‐β isoforms.

Cys77 or Cys109 in TGF‐β isoforms is important to form homodimer for activation of their signalling activities.^25^, ^26^ In HCV‐infected Huh7.5 cells, Cys77 substituted with serine or Cys109 exchanged by tyrosine completely eliminated the anti‐HCV activities of TGF‐β1 and TGF‐β3 (Figure 4B,C), indicating that dimer formation mediated by Cys77 or Cys109 residues was responsible for the anti‐HCV activities of TGF‐β isoforms.

The amino acid residues Arg25, Val92 and Arg94 of TGF‐β1 and TGF‐β3 are responsible for their high‐affinity interaction with TβRII, once they are exchanged with Lys25, Ile92 and Lys94, respectively, TGF‐β signalling activities of TGF‐β1 and TGF‐β3 are reduced.^27^ Though TGF‐β1 and TGF‐β3 significantly inhibited HCV propagation, TGF‐β isoforms with mutants at R25K, V92I and R94K inversely promoted HCV propagation when the plasmids were introduced into HCV‐infected Huh7.5 cells (Figure 4D). It was surprised that Cys77 or Cys109 residues, or Arg25, Val92 and Arg94 mutants of TGF‐β1 and TGF‐β3, on the contrary, promoted HCV propagation (Figure 4B‐D). Because of the significant sequence homology of TGF‐β isoforms (Figure 1A) and their possibly direct interaction with HCV (Figure 2C), it might be that the conformation of the mutants would contribute to the direct interaction to facilitate HCV entry into cells. However, the mechanism needs to be further investigated.

The α‐helix 3 of TGF‐β isoforms is response for their binding surface for TβRI and truncating α‐helix 3 of TGF‐β2 suppresses its signalling activity.^28^ However, loss of α‐helix 3 of TGF‐β1 and TGF‐β3 with deletion of residues 52‐71 aa did not interfere with their anti‐HCV activities (Figure 4D). These results indicated the truncated α‐helix 3 might partly block TGF‐β/SMAD signalling activities of TGF‐β1 but was insufficient to influence the anti‐HCV activities of TGF‐β1 and TGF‐β3, or there was other antiviral mechanism independent of TGF‐β/SMAD signalling.

Interestingly, by blocking the TGF‐β receptor kinase activity or silencing SMAD2/3, we presumed the anti‐HCV activities of TGF‐β1 and TGF‐β2 might be through TGF‐β/SMAD signalling pathway, while TGF‐β3 inhibited HCV replication independent on TGF‐β/SMAD signalling pathway (Figure 3). However, blocking TGF‐β1/3 dimerization or the ligand‐receptor II interaction led to abolishment of TGF‐β1/3‐mediated HCV propagation (Figure 4). This discrepancy might highlight that although TGF‐β3 inhibited HCV replication independent on TGF‐β/SMAD signalling pathway. The amino acid sequences, which were responsible for TGF‐β3 homodimer formation or the ligand‐receptor II interaction, were also important to the anti‐HCV effects of TGF‐β3.

### 3.5 TGF‐β1 and TGF‐β2, except TGF‐β3, mediate HCV promoting hepatic stellate cell activation

**FIGURE 5:**
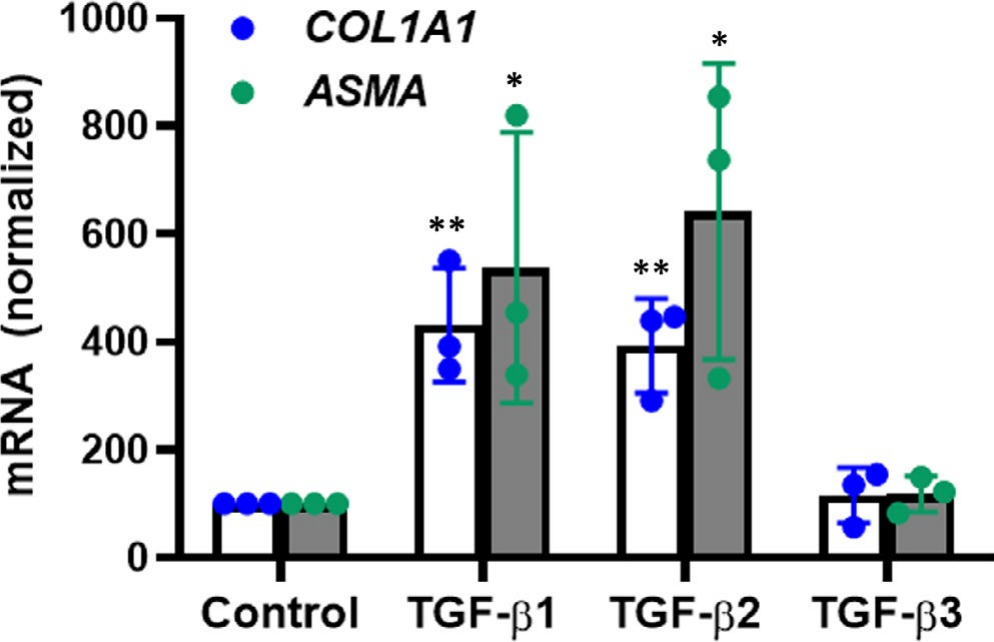
TGF‐β1 and TGF‐β2, except TGF‐β3, promote hepatic stellate cell activation. LX‐2 cells were stimulated with 0.5 nmol/L TGF‐β isoforms for 24 h and *COL1A1* and *α‐SMA* mRNA was quantified with qRT‐PCR. Data represent the mean ± SD of three independent experiments. ANOVA analysis followed by the Student’s *t* test method was used. **P < 0.01 vs solvent control

Activation of TGF‐β1 initiates liver fibrotic tissue response, a programme of temporary collagen accumulation, and then induces pathogenic fibrosis.^29^, ^30^ We thus evaluated whether the TGF‐β isoforms promoted hepatic stellate cell activation at their antiviral effective concentrations. Indeed, 0.5 nmol/L of TGF‐β1 and TGF‐β2 which concentration was active against HCV propagation, significantly increased *COL1A1* and *α‐SMA* mRNA expression (Figure 5), while 0.5 nmol/L of TGF‐β3 did not enhance *COL1A1* and *α‐SMA* mRNA expression in LX‐2 cells (Figure 5).

## 4 DISCUSSION

In this study, we found all of the three TGF‐β isoforms suppressed HCV propagation through interrupting several different stages in the whole life cycle of HCV, including virus entry and intracellular replication stages, in TGF‐β/SMAD signalling pathway–dependent and TGF‐β/SMAD signalling pathway–independent manners, and therefore, they had additional anti‐HCV activities.

Virus infection, particularly at the early stage, triggers the early host antiviral defence and adaptive immunity.^31^, ^32^ Hence, it commonly induces chronic inflammation, which is a leading cause of chronic disease, such as HCV infection induced fibrosis, cirrhosis and even liver cancer.^33^, ^34^ In the disease progression, TGF‐β isoforms play crucial roles in the pro‐inflammatory and anti‐inflammatory response process.^4^, ^35^, ^36^ In healthy individuals, the levels of TGF‐β isoforms in serum are different. TGF‐β1 exhibits the highest level with about dozens of ng/mL, while TGF‐β2 and TGF‐β3 are relative low,^5^, ^37^, ^38^ with the median concentration of TGF‐β3 in plasma about 189 pg/mL (0.00756 nmol/L).^39^ However, once HCV infection, TGF‐β isoforms were up‐regulated in both serum and liver, with the concentration of TGF‐β1 at 87.5 ng/mL (3.5 nmol/L) and TGF‐β2 at 0.3 ng/mL (0.012 nmol/L) in serum.^5^, ^6^, ^37^, ^40^ In HCVcc system, we also demonstrated the elevated expression of the three TGF‐β isoforms after HCV infection. However, the elevated TGF‐β isoforms, in turn, inhibited HCV propagation. Previous studies have reported that TGF‐β can inhibit HCV RNA replication in HCV sub‐genomic replicon system and also directly restrict HBV replication in the cell culture system.^10^, ^11^ We also demonstrated that all of the three TGF‐β isoforms could suppress HCV propagation in HCVcc system. Most importantly, the three TGF‐β isoforms could additionally inhibit HCV propagation under the pathophysiological condition. TGF‐β/SMAD signalling pathway activation could contribute to hepatic stellate cells activation, collagen gene transcription and liver fibrosis and was associated with later tumour progression.^41^, ^42^ Previous studies confirmed that chronic inflammation associated with HCV infection perturbed hepatic TGF‐β signalling and then promoted fibrogenesis, and TGF‐β2 released from HCV‐infected cells contributed to hepatic fibrogenic responses through TGF‐β signalling.^5^, ^43^ Our results also demonstrated TGF‐β1 and TGF‐β2 could induce the expression of *COL1A1* and *α‐SMA* in LX‐2 cells at their corresponding antiviral concentrations, suggesting that TGF‐β1 and TGF‐β2 might have dual functions in HCV infection, including anti‐HCV activities and promoting fibrosis effects through activating TGF‐β signalling.

All of the three TGF‐β isoforms suppressed HCV propagation. However, they inhibited HCV propagation at different stages of HCV life cycle. TGF‐β1 inhibited HCV propagation in the whole viral life cycle, including before, during and after virus entry, and intracellular HCV replication steps. Both of TGF‐β2 and TGF‐β3 targeted infectious HCV particle inactivation and intracellular HCV replication. But except TGF‐β3, pre‐treatment of TGF‐β2 for 2 hours before HCV infection also inhibited HCV propagation after virus entry stage. Furthermore, we found all of the three TGF‐β isoforms could reduce *de novo* produced HCV without inhibiting HCV release step. The detailed mechanisms showed that the anti‐HCV effects of TGF‐β1 and TGF‐β2, except TGF‐β3, were partly dependent on activating TGF‐β/SMAD signalling pathway by applying the inhibitors and siRNA focusing on TGF‐β/SMAD signalling pathway. The results from amino acids mutant experiments also suggested that TGF‐β signalling activated by TGF‐β1 was critical for its anti‐HCV effect. Although TGF‐β1 and TGF‐β2 suppressed HCV propagation dependent on TGF‐β/SMAD signalling, they inhibited HCV in different HCV infection stages, which might be owing to their different TβRII affinity and subsequently anti‐HCV gene expression.^44^, ^45^ In addition, TGF‐β isoforms might inhibit HCV propagation in a TGF‐β/SMAD signalling pathway–independent manner because they were still active against HCV propagation when the α‐helix 3 of TGF‐β isoforms was deleted. Other antiviral mechanisms of TGF‐β isoforms remain to be clarified. Furthermore, owing to part of anti‐HCV mechanisms of TGF‐β isoforms was the same, the additional anti‐HCV activities might depend on both of promoting the same mechanism and acting on different mechanisms.

In conclusion, HCV infection increases the level of TGF‐β isoforms in infected hepatocytes, and thus, the elevated TGF‐β1 and TGF‐β2 exacerbate the liver disease, such as fibrosis. In turn, the elevated TGF‐β isoforms might be one of the host antiviral defenders through interrupting HCV life cycle in TGF‐β/SMAD signalling pathway–dependent and TGF‐β/SMAD signalling pathway–independent manners.

## CONFLICT OF INTEREST

The authors declare no conflicts of interest.

## AUTHOR CONTRIBUTIONS

**Li‐Li Zou:** Conceptualization (equal); Data curation (lead); Methodology (lead); Writing‐original draft (equal); Writing‐review & editing (lead). **Jian‐Rui Li:** Data curation (lead); Methodology (lead). **Hu Li:** Methodology (supporting); Writing‐review & editing (supporting). **Jia‐Li Tan:** Data curation (supporting); Methodology (supporting); Writing‐review & editing (supporting). **Mei‐Xi Wang:** Writing‐review & editing (supporting). **Nan‐Nan Liu:** Writing‐review & editing (supporting). **Rong‐Mei Gao:** Data curation (supporting); Methodology (supporting). **Hai‐Yan Yan:** Data curation (supporting); Methodology (supporting). **Xue‐Kai Wang:** Writing‐review & editing (supporting). **Biao Dong:** Methodology (supporting). **Yu‐Huan Li:** Methodology (supporting). **Zonggen Peng:** Conceptualization (lead); Data curation (lead); Formal analysis (lead); Funding acquisition (lead); Methodology (lead); Project administration (lead); Writing‐review & editing (lead).

## Data Availability

The data used to support the findings of this study are available from the corresponding author upon request.
